# Association of Low-Risk Human Papillomavirus Infection with Male Circumcision in Young Men: Results from a Longitudinal Study Conducted in Orange Farm (South Africa)

**DOI:** 10.1155/2011/567408

**Published:** 2011-04-07

**Authors:** Chloé Tarnaud, Pascale Lissouba, Ewalde Cutler, Adrian Puren, Dirk Taljaard, Bertran Auvert

**Affiliations:** ^1^CESP, INSERM-UVSQ, UMRS 1018, Hôpital Paul Brousse, 16 avenue Paul Vaillant-Couturier, 94807 Villejuif Cedex, France; ^2^National Institute for Communicable Diseases (NICD), National Health laboratory Services (NHLS), Private Bag X4, Sandringham 2131, South Africa; ^3^Faculty of Health Sciences, University of the Witwatersrand, Private Bag 3, Wits 2050, South Africa; ^4^Progressus, Postnet Suite 51, Private Bag X1, 2115 Northcliff, South Africa; ^5^UMRS1018, Versailles Saint-Quentin-en-Yvelines University, 78035 Versailles, France; ^6^Hôpital Ambroise Paré, Assistance Publique-Hôpitaux de Paris, 9, avenue Charles-de-Gaulle, 92100 Boulogne Billancourt, France

## Abstract

*Background*. Low-Risk Human Papillomavirus (LR-HPV) genotypes 6 and 11 cause genital warts. This study investigated the association of LR-HPV infection with male circumcision (MC). *Methods*. We used data from the South African MC trial conducted among young men. Urethral swabs, collected among intervention (circumcised) and control (uncircumcised) groups, were analyzed using HPV linear array. Adjusted LR-HPV prevalence rate ratio (aPPR) and Poisson mean ratio (aPMR) of number of LR-HPV genotypes were estimated using log-Poisson regression, controlling for background characteristics, sexual behaviour, and HIV and HSV-2 statuses. *Results*. Compared to controls, LR-HPV prevalence and mean number of genotypes were significantly lower among the intervention group ((8.5% versus 15.8%; aPRR: 0.54, *P* < .001) and (0.33 versus 0.18; aPMR: 0.54, *P* < .001), resp.). Mean number of LR-HPV genotypes increased with number of lifetime sexual partners and decreased with education level and consistent condom use. *Conclusions*. This study shows a reduction in LR-HPV infection among circumcised men.

## 1. Introduction

Human Papillomavirus (HPV), a small DNA virus, is the most common sexually transmitted infection in the world. HPV prevalence in Africa is disproportionably high, ranging from 22.1% to 46.0% among women [1–3]. More than 100 HPV genotypes have been identified, of which about 40 infect the mucosal epithelium. They are divided into “high-risk” and “low-risk” genotypes on the basis of their association with malignant lesions of the cervix. High-risk HPV (HR-HPV) infection has been linked with genital cancer [4–6].

Low-risk HPV (LR-HPV) genotypes 6 and 11 cause around 90% of genital warts (GWs), also called condylomata acuminate [7]. In Sub-Saharan Africa, LR-HPV prevalence has been estimated at 13.6% [2], ranging from 1.2% to 30.0% depending on geographical region, target population, and sexual behaviour [3]. Although treatment helps to eliminate the virus, GWs are often painful, and new lesions or recurrences are frequent, resulting in patient dissatisfaction and leading to psychological distress [7, 8]. GWs are associated with a significant decline in health-related quality of life [9]. A study assessing the psychological burden of HPV-related diseases found that GWs had the largest impact on quality of life compared with other clinical forms, chiefly due to their impact on sexuality, self-image, and partner transmission [10]. 

There is evidence that multiple HPV infection may have important clinical and epidemiological implications. Infection with multiple HPV genotypes has been associated with concurrent anogenital warts [11–13], acquisition of new HPV type and HPV infection persistence [14], dysplasia [15], cervical intraepithelial neoplasia severity [16, 17], cytological abnormalities in anal specimens [18], and HIV coinfection [19–21]. 

Hence, the analysis of LR-HPV infection in terms of number of LR-HPV genotypes detected in urethral swabs is important since being infected with one genotype may be different from being infected with two or more genotypes.

A recent observational study has suggested that HPV prevalence was reduced among circumcised men compared to uncircumcised men [22], whereas other studies failed to find an association [23, 24]. However, two recent male circumcision (MC) trials have demonstrated a lower HR-HPV prevalence and incidence among circumcised men [25–27]. This protective effect could be explained by the role of the foreskin on HPV transmission [28]. Consequently, the same protective effect of MC can be expected on LR-HPV prevalence, but to date, very little is known about this association. 

The primary objective of this study was to investigate the effect of MC on LR-HPV infection by considering both LR-HPV prevalence and the number of LR-HPV genotypes detected among young men. The secondary objective was to identify other risk factors of LR-HPV infection. Data used were collected during the MC randomized controlled trial (RCT) conducted in Orange Farm (South Africa), which demonstrated a partially protective effect of MC on the acquisition of HIV [29].

## 2. Methods

### 2.1. Collection of Data

Technical details of the Orange Farm MC RCT (ANRS-1265) have been published elsewhere [29]. The trial took place in Orange Farm, a semiurban township neighbouring the city of Johannesburg, between February 2002 and July 2004. A total of 3274 uncircumcised men, aged 18 to 24, were recruited from the general population. They were randomly assigned to be either immediately circumcised (intervention group) or to undergo circumcision after the end of the trial (control group) and were followed up for 21 months. During each of the four visits (at baseline and follow-up visits at months 3, 12, and 21), circumcision status was assessed by a trained nurse via genital examination, blood samples were obtained and tested for HIV as well as HSV-2, and a face-to-face questionnaire was administered. 

The current study uses data collected during the 21-month visit. Participants were included in the trial regardless of their HIV and HSV-2 status. The sample includes all the men, among the trial participants, who reported for the 21-month follow-up visit between March 7th, 2005 and November 24th, 2005, and among whom urethral swabs were collected during this period. The selection has been described in further details in a previous publication [25]. Five hundred and ninety six follow-up visits were added to the original database analyzed to report results on the association between MC status and HR-HPV [25], yielding a total of 1768 follow-up visits. One urethral swab was collected by the same nurse from each participant and tested for HPV. All participants signed a written consent form for this test to be performed. The collection of swabs could not be started earlier because of limited funding. Swabs were analyzed to assess the association between MC and both prevalence and number of LR-HPV genotypes. The 21-month questionnaire allowed for data collection on background characteristics (age, ethnic group, level of education, number of lifetime sexual partners, and marital status) as well as reported sexual behaviour in the past 12 months (number of sexual contacts and frequency of condom use).

### 2.2. Laboratory Methods

Detailed laboratory methods for HIV-1 and HSV-2 testing have previously been reported [29, 30]. Urethral swabs were frozen at −20°C immediately after collection and kept frozen until processing. DNA was extracted from the specimens using the MagNA Pure LC (Roche) instrument, with the Roche MagNA Pure LC DNA I Isolation Kit. Swabs were lysed in 500 *μ*L of the kit lysis buffer for 30 minutes at room temperature. The MagNa Pure external lysis protocol was used to extract DNA from the lysis buffer into a 100 *μ*L eluate. 50 *μ*L of the eluate was used for screening (Roche Amplicor HPV test), and the other 50 *μ*L eluate was used for genotyping (Roche Linear Array Genotyping test). This standardized PCR-based method can detect 37 genotypes of HPV [31, 32]. Fifteen results (15/1768; 0.84%) with a negative internal beta globin PCR control were excluded, four in the control arm (4/867; 0.46%) and eleven in the intervention arm (11/901; 1.2%). All positives were genotyped. An LR-HPV-positive sample was defined as a sample where at least one LR-HPV genotype was detected.

### 2.3. Data Analysis

Roche Linear Array Genotyping test was used to detect 23 HPV genotypes which are classified as nononcogenic. In this paper, all of the following 23 genotypes are characterized as LR-HPV: 6, 11, 26, 40, 42, 53, 54, 55, 61, 62, 64, 67, 69, 70, 71, 72, 73, 81, 82, 83, 84, IS39, and CP6108. A more conservative approach was used, which considered a subset of 11 genotypes (i.e., genotypes: 6, 11, 26, 40, 42, 53, 54, 55, 83, 84, CP6108), excluding any genotype having been labelled as of intermediate risk in the literature or not listed in the LR-HPV genotypes list [5, 33–35].

We used multivariate log-Poisson regression to analyze both LR-HPV positivity and number of LR-HPV genotypes detected for each participant. Results are formulated in terms of prevalence rate ratios (PRRs) of LR-HPV positivity and Poisson mean ratio (PMRs) of the mean number of LR-HPV genotypes, respectively, comparing men of the intervention group with men of the control group. The intention-to-treat (ITT) and as-treated (AT) adjusted PRRs (aPRRs) and PMRs (aPMRs) were estimated controlling for the following covariates: ethnic group, age, education, number of lifetime sexual partners, condom use in the past 12 months, as well as HIV and HSV2 statuses at the 21-month visit. Analyses were repeated using as an outcome the subset of 11 LR-HPV genotypes. Because HIV is reduced by MC and is associated with HPV infection [36], we assessed the potential impact of HIV acquisition on the results by excluding those who HIV seroconverted during the follow-up period. Finally, to evaluate the effect of a possible imbalance between the groups, analyses were repeated using propensity scores coded in quintiles [37]. Percentages were compared with Fisher's exact test and means with the Mann-Whitney test. Statistical analyses were performed using STATA 9.0 (StataCorp. 2005. Stata Statistical Software: Release 9. College Station, TX: StataCorp LP).

### 2.4. Ethics

The trial was conducted with the understanding and the written consent of all participants. The research protocol was reviewed and approved by the University of the Witwatersrand Human Research Ethics Committee (Medical) on February 22nd, 2002 (protocol study no. M020104). The trial was also approved by the Scientific Commission of the French National Agency for AIDS Research (ANRS; protocol study no. 1265; 2002, decision no. 50), and authorization was obtained from the City of Johannesburg, Region 11, on 25 February 2002. This trial has been registered at http://www.clinicaltrials.gov/ under the number NCT00122525.

## 3. Results

The 1753 participants from whom a urethral swab was collected at the 21-month visit and analysed were included in the analysis. The mean (median) durations in days of follow-up among the intervention and control group were 653 (637) and 648 (637), respectively. 

Table 1 reports the study population's baseline characteristics, reported sexual behaviour and HIV, HSV-2, and LR-HPV prevalences at the 21-month visit. Median age was 21.2 years (mean: 21.2, 95%CI: 21.2–21.3). The most frequent sexually transmitted infection (STI) was LR-HPV. Compared to control group participants, men from the intervention group were slightly older (mean: 21.3 versus 21.1, *P* = .04) and reported more frequently having had a sexual partnership in the last year. Level of education, living with a partner, number of lifetime sexual partners, and consistent condom use were not statistically different between the two groups. HIV and LR-HPV prevalences at the 21-month visit were significantly lower in the intervention group in comparison with the control group. The mean number of LR-HPV genotypes was 0.25 (95%CI: 0.21–0.30).

**Figure 1:**
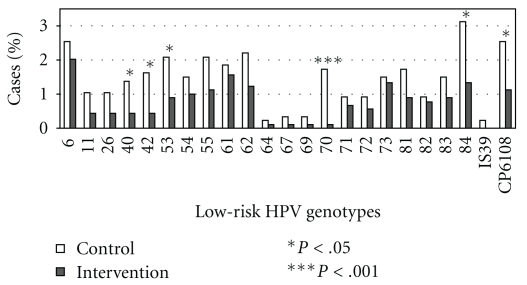
Distribution of the low-risk HPV genotypes as a function of randomization group among the 1753 participants (intention to treat population).

**Figure 2:**
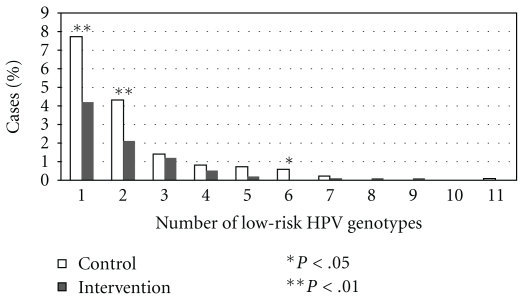
Distribution of the number of low-risk HPV genotypes collected in each urethral swabs as a function of randomization group among the 1753 participants (intention to treat population).

The number of HR-HPV and LR-HPV genotypes were correlated (*ρ* = 0.67, *P* < .001). Among HR-HPV-infected men, 56.2% (95%CI: 50.7–61.6) were infected with LR-HPV. Among LR-HPV-infected men, 85.9% (95%CI: 81.1–90.6) were infected with HR-HPV.  Figure 1 indicates that the percentage of each of the 23 LR-HPV genotypes was always lower among men from the intervention group compared to men from the control group. In the ITT comparison, the difference was significant for genotypes 40, 42, 53, 70, 84, and CP6108. Among the 212 LR-HPV-infected men, 51.4% (95%CI: 44.6–58.2) were infected with more than one LR-HPV genotype, for a maximum of 11 genotypes. Figure 2 shows the distribution of the number of LR-HPV genotypes among LR-HPV-infected participants.

### 3.1. Risk Factors for Urethral LR-HPV Infection

The prevalence of LR-HPV infection was significantly lower among intervention group participants compared to control group participants (8.5% versus 15.8%; ITT aPRR: 0.54, 95%CI: 0.41–0.72, *P* < .001 and AT aPRR: 0.53, 95%CI: 0.40–0.70, *P* < .001).

Table 2 shows the univariate and multivariate PMRs of the number of LR-HPV genotypes by MC status at the scheduled 21-month visit. In both univariate and multivariate ITT and AT analyses, the mean number of LR-HPV genotypes in the intervention group was significantly lower, about half that of the control group. The association was slightly stronger in the AT analyses. 

Results remained unchanged when the more restrictive definition of 11 LR-HPV genotypes was considered, with a twofold decrease of the mean number of LR-HPV in the intervention group compared with the control group (0.10 versus 0.21; ITT aPMR: 0.51, 95%CI: 0.39−0.66, *P* < .001).

Mean number of LR-HPV genotypes significantly increased with the number of lifetime sexual partners (ITT aPMR: 1.05, 95%CI: 1.02–1.09, *P* = .001). Prevalent HIV and HSV-2 infections at the 21-month visit were associated with an increased number of LR-HPV genotypes (ITT aPMR: 3.40, 95%CI: 2.65–4.36, *P* < .001 and ITT aPMR: 1.49, 95%CI: 1.15–1.92, *P* = .002, resp.). Conversely, mean number of LR-HPV genotypes was lower for participants having completed the primary level of education (ITT aPMR: 0.47, 95%CI: 0.28–0.79, *P* = .004) and those reporting consistent condom use (ITT aPMR: 0.65, 95%CI: 0.45–0.93, *P* = .02). 

When participants who HIV seroconverted during follow-up (*n* = 33) were excluded from the analyses, the effect of MC on number of LR-HPV remained unchanged (ITT aPMR: 0.53, 95%CI: 0.43–0.65, *P* < .001, and AT aPMR: 0.47, 95%CI: 0.38–0.58, *P* < .001). This implies that the effect of MC on LR-HPV is independent of the effect of MC on HIV.

The aPMRs were almost identical when the analyses were adjusted for the propensity score in addition to the other covariates (ITT aPMR: 0.55, 95%CI: 0.45–0.66, *P* < .001, and AT aPMR: 0.46, 95%CI: 0.38–0.57, *P* < .001). This suggests that a possible imbalance between groups due to a differential follow-up had no effect on the association of LR-HPV infection with MC.

## 4. Discussion

Using data collected during the MC trial conducted in Orange Farm (South Africa), we found a strong independent association between LR-HPV urethral infection and MC. LR-HPV infection was analysed as a prevalence rate and in terms of number of LR-HPV detected in urethral swabs, because it was assumed that being infected with one genotype was different from being infected with two or more genotypes [12, 13, 20]. It was found that both risks of being infected with one LR-HPV genotype (PMR) and with at least one genotype (PPR) were consistently decreased among men from the circumcised group. These men did not differ significantly from control group participants in terms of sexual behaviour, apart from reporting more frequently at the 21-month visit having had at least one sexual partnership in the past 12 months.

The mechanisms by which circumcised men are less likely to be infected with HPV could be due to a reduced acquisition of new infection or an increased clearance of pre-existing infection, because the absence of foreskin may reduce the risk of autoreinfection at the urethral site [28]. 

This study has some limitations, primarily the lack of data on HPV status at baseline, so the causality of MC on reduced LR-HPV infection cannot be rigorously demonstrated. However, MC was randomly assigned, and controlling for the propensity score didnot affect the results. A second limitation is that participants were not blinded, so they might have changed their sexual behaviour according to their randomization group. Lastly, detection of LR-HPV was performed on urethral swabs, which are known to miss infections compared with detection in the glans, corona sulcus, or penile shaft [38, 39]. Thus, the prevalence of LR-HPV in our cohort may be underestimated, but such underestimation would be equally distributed among the two groups of randomization. Hence, its impact on PMR is likely to be small, and despite this potential loss of power, our results evidenced a significant protective effect of MC on urethral LR-HPV infection.

To date, little was known on the association of MC with LR-HPV. Few observational studies have studied this association but results remained inconsistent [22, 23, 40, 41]. However, Tobian and colleagues [27] have recently published results similar to those of this study, analyzing data collected in another African population and using a different HPV swabbing site. They reported a 44% reduction in LR-HPV prevalence among circumcised men. Hence, we can conclude that the protective effect of MC on HR-HPV infection can be generalized to LR-HPV genotypes. 

In accordance with observational studies, our study reveals some other risk factors of LR-HPV, such as the number of lifetime sexual partners [22–24, 42, 43] and being older than 21 years old [14], although the role of age may vary [22, 43]. Lastly, our results bring evidence supporting the protective role of condom use on HPV and LR-HPV infections [14, 23, 40, 43].

## 5. Conclusion

MC, an immediately available and cost-effective method, was proven to reduce HR-HPV infection [25, 27]. Hence the protective effect of MC on LR-HPV, demonstrated in this study, provides new arguments endorsing the WHO-UNAIDS recommendation for the implementation of MC programs in targeted countries [44].

## Figures and Tables

**Table 1:** Background characteristics, reported sexual behaviour, and HIV, HSV-2 and low-risk HPV prevalence of the study population.

	Control *N* = 863	Intervention *N* = 890	*P* (1)
Background Characteristics			
Ethnic group (%) Sotho Zulu Other	53.2 33.1 13.7	54.2 28.3 17.5	.02
Lives with a partner (%) (2)	4.7	5.5	.61
Aged 21 and older (%)	50.1	55.1	.04
Primary level of education completed (%)	98.8	(98.0)	.18

Reported sexual behaviour			
Mean (median) number of lifetime partners	4.3 (4.0)	4.4 (4.0)	.33
Had at least one sexual partnership in the past 12 months (%)	71.2	77.9	.004
Mean (median) number of partnerships in the past 12 months (2)	1.4 (1.0)	1.3 (1.0)	.70
Mean (median) number of sexual intercourses in the past 12 months (2)	13.3 (5.0)	13.9 (6.0)	.41
Consistent condom use in the past 12 months (%) (2)	23.6	24.9	.70

Sexually transmitted infection prevalence at the 21-month visit			
HIV positive (%)	7.2	4.6	.03
HSV-2 positive (%)	8.6	10.6	.17
Low-risk HPV positive (23 genotypes) (%)	15.8 (*n* = 136)	8.5 (*n* = 76)	<.001
Low-risk HPV positive (11 genotypes) (%)	12.4 (*n* = 107)	6.6 (*n* = 59)	<.001

(1) Fisher's exact for percentage comparison and Mann-Whitney test for mean comparison;

(2) among those having at least one sexual intercourse in the past 12 months (437 control and 497 intervention participants).

**Table 2:** Intention-to-treat (ITT) and As-treated (AT) Poisson Mean Ratios (PMRs) of the number of low-risk HPV (LR-HPV) genotypes among the 1753 participants.

	Number of participants (LR-HPV infected)	Mean of LR-HPV genotypes	PMR (95% CI)	ITT aPMR (1) (95% CI)	AT aPMR (1) (95% CI)
Circumcision status					
Randomization group Control Intervention	863 (136) 890 (76)	0.33 0.18	1 0.53 (0.44–0.64) *P* < .001	1 0.54 (0.44–0.66) *P* < .001	not applicable
Circumcision status Uncircumcised Circumcised	855 (136) 898 (76)	0.35 0.16	1 0.45 (0.37–0.55) *P* < .001	not applicable	1 0.46 (0.37–0.56) *P* < .001

Background characteristics					
Ethnic group Sotho Zulu Other	941 (116) 538 (66) 274 (30)	0.24 0.27 0.26	1 1.12 (0.91–1.37) *P* = .29 1.05 (0.80–1.37) *P* = .75	1 1.05 (0.86–1.30) *P* = .63 1.00 (0.76–1.30) *P* = .98	1 1.04 (0.84–1.28) *P* = .77 1.06 (0.81–1.39) *P* = .56
Age Under 21 21 and older	831 (82) 922 (130)	0.20 0.30	1 1.53 (1.26–1.85) *P* < .001	1 1.08 (0.88–1.32) *P* = .48	1 1.10 (0.90–1.35) *P* = .27
Primary level of education Uncompleted Completed	28 (6) 1725 (206)	0.54 0.25	1 0.47 (0.28–0.78) *P* = .004	1 0.50 (0.30–0.84) *P* = .009	1 0.48 (0.29–0.81) *P* = .006

Reported sexual behaviour					
Number of lifetime sexual partners two or less more than two	586 (49) 1167 (163)	0.14 0.31	1 2.24 (1.76–2.84) *P* < .001	1 1.68 (1.30–2.16) *P* < .001	1 1.69 (1.31–2.18) *P* < .001
Condom use in the past 12 months^(2)^ Inconsistent Consistent	695 (99) 223 (19)	0.32 0.15	1 0.48 (0.33–0.68) *P* < .001	1 0.66 (0.46–0.95) *P* = .03	1 0.68 (0.47–0.99) *P* = .04

Sexually transmitted infection prevalence at the 21-month visit					
HIV negative positive	1650 (175) 103 (37)	0.21 1.05	1 5.10 (4.11−6.34) *P* < .001	1 3.29 (2.57–4.22) *P* < .001	1 3.20 (2.49–4.10) *P* < .001
HSV-2 negative positive	1585 (173) 168 (39)	0.22 0.59	1 2.68 (2.15−3.36) *P* < .001	1 1.49 (1.15–1.93) *P* = .002	1 1.52 (1.17–1.96) *P* = .002

(1) Adjusted for ethnic group, age, education, number of lifetime sexual partners, condom use in the past 12 months, and HIV and HSV2 statuses at the 21-month visit;

(2) among those having at least one sexual intercourse in the past 12 months.
